# The interaction effect of transfusion history and previous stroke history on the risk of venous thromboembolism in stroke patients: a prospective cohort study

**DOI:** 10.1186/s12959-023-00487-2

**Published:** 2023-04-17

**Authors:** Changqing Sun, Rongrong Wang, Lianke Wang, Panpan Wang, Ying Qin, Qianyu Zhou, Yuanli Guo, Mingyang Zhao, Wenqian He, Bo Hu, Zihui Yao, Peijia Zhang, Tiantian Wu, Yu Wang, Qiang Zhang

**Affiliations:** 1 School of Nursing and Health, Zhengzhou University, 101 Kexue Avenue, Zhengzhou, 450001 Henan PR China; 2 School of Public Health, Zhengzhou University, Zhengzhou, PR China; 3 Department of Neurology, the First Affiliated Hospital of Zhengzhou University, Zhengzhou, PR China

**Keywords:** Interaction, Previous stroke history, Stroke, Transfusion history, Venous thromboembolism

## Abstract

**Background:**

Blood transfusion and previous stroke history are two independent risk factors of venous thromboembolism (VTE) in stroke patients. Whether the potential interaction of transfusion history and previous stroke history is associated with a greater risk of VTE remains unclear. This study aims to explore whether the combination of transfusion history and previous stroke history increases the risk of VTE among Chinese stroke patients.

**Methods:**

A total of 1525 participants from the prospective Stroke Cohort of Henan Province were enrolled in our study. Multivariate logistic regression models were used to explore the associations among transfusion history, previous stroke history and VTE. The interaction was evaluated on both multiplicative and additive scales. The odds ratio (95% CI), relative excess risk of interaction (RERI), attributable proportion (AP), and synergy index (S) of interaction terms were used to examine multiplicative and additive interactions. Finally, we divided our population into two subgroups by National Institutes of Health Stroke Scale (NIHSS) score and re-evaluated the interaction effect in both scales.

**Results:**

A total of 281 (18.4%) participants of 1525 complicated with VTE. Transfusion and previous stroke history were associated with an increased risk of VTE in our cohort. In the multiplicative scale, the combination of transfusion and previous stroke history was statistically significant on VTE in both unadjusted and adjusted models (*P*<0.05). For the additive scale, the RERI shrank to 7.016 (95% CI: 1.489 ~ 18.165), with the AP of 0.650 (95% CI: 0.204 ~ 0.797) and the S of 3.529 (95% CI: 1.415 ~ 8.579) after adjusting for covariates, indicating a supra-additive effect. In subgroups, the interaction effect between transfusion history and previous stroke history was pronouncedly associated with the increased risk of VTE in patients with NIHSS score > 5 points (*P*<0.05).

**Conclusions:**

Our results suggest that there may be a potential synergistic interaction between transfusion history and previous stroke history on the risk of VTE. Besides, the percentage of VTE incidence explained by interaction increased with the severity of stroke. Our findings will provide valuable evidence for thromboprophylaxis in Chinese stroke patients.

**Supplementary Information:**

The online version contains supplementary material available at 10.1186/s12959-023-00487-2.

## Introduction

Venous thromboembolism (VTE), consisting of deep vein thrombosis (DVT) and pulmonary embolism (PE), is one of the common but preventable complications of stroke patients [1]. VTE is characterized by a high incidence rate, high mortality, and heavy economic health­care burden despite advances in diagnosis and treatment [2]. The incidence of VTE in stroke patients can be as high as 21.1%-28.5%, 40% of stroke patients can develop VTE in the first 3 weeks, and the mortality even can be up to 16.70% within 30 days [3–5].

Previous studies have shown that a transfusion history (TH) could increase the risk of VTE [6, 7]. Transfused red blood cells have been proposed to modulate the inflammatory cascade [8]. This could result from a direct immune response to the blood transfusion or oxidative stress related to the storage of red blood cells and consequently surface damage resulting in a pro-coagulative state [9]. Besides, a history of transfusion was included as a risk factor in the Caprini score of 2013 version [10].

Additionally, previous studies have also confirmed that patients with a previous stroke history (PSH) were more likely to have provoked VTE than those without stroke, and patients with VTE who had a prior stroke were more than twice as likely to die while hospitalized and within 30 days of VTE diagnosis compared to those without previous stroke [11]. Patients with a prior stroke may be more prone to thrombosis due to the impairment of limb movement and hypercoagulability [12].

According to the pathophysiology and Virchow’s triad theory of thrombosis, the blood was hypercoagulable in patients with a history of stroke and transfusion [13]. Therefore, it is reasonable to speculate that coagulation abnormalities arising from two distinct acquired sources could increase the risk of VTE. Previous interaction studies in the field mainly focused on the effect of gene-gene interaction on thrombosis [14, 15]. The interaction effect between genetics and environmental factors on VTE incidence has also been shown [16]. A few studies have explored the joint effect of individual factors including obesity, smoking, cancer and other factors that were associated with an increased VTE risk [17, 18]. However, the interaction effect between transfusion history and prior stroke history on VTE in stroke patients is unclear. Exploring and understanding the possible associations between the two risk factors are of great significance for thromboprophylaxis in stroke patients.

Therefore, our study aims to evaluate whether there is an interaction effect between the history of blood transfusion and prior stroke on VTE in a large prospective cohort of Chinese stroke patients.

## Materials and methods

### Study participants

**Fig. 1:**
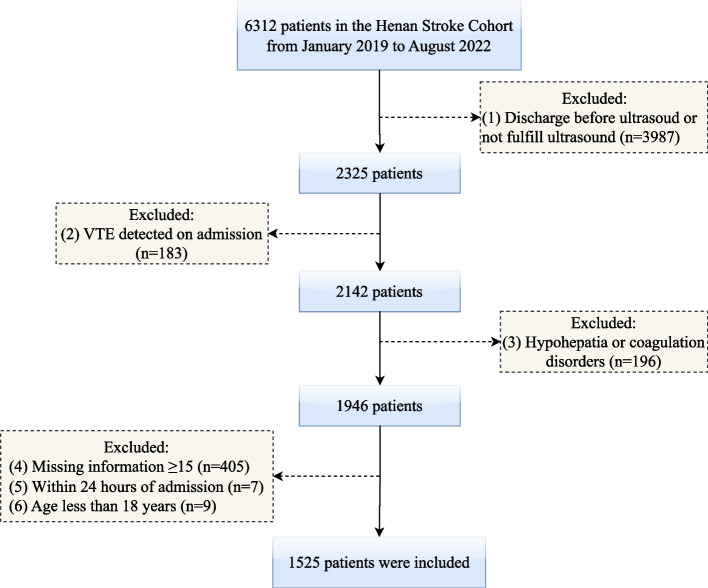
Study flowchart

The participants of this study were derived from the Stroke Cohort of Henan Province, an ongoing prospective study of 11072 stroke patients aged 18 or older from 2011 up to now. Henan is the third largest province with a population of about 100 million in China, which is sufficient to represent the diversity of the Chinese population. All the participants completed an interviewer-assisted comprehensive questionnaire to collect baseline information, including demographic characteristics, lifestyle factors, laboratory and imaging examination results. Also, the information can be linked to electronic health records. The inclusion criteria of participants were: (a) the patients were diagnosed with stroke by neurological specialists and confirmed by brain computed tomography scan or magnetic resonance imaging; (b) aged 18 years and older; and (c) patients with definite imaging examination of blood vessels of extremities by color doppler ultrasonography or venography, no history of VTE at baseline, and successfully followed up until be discharged from hospital. The exclusion criteria were: (a) patients were discharged within 24 hours; (b) other systemic diseases leading to coagulation abnormalities, such as cirrhosis, cardiolipin syndrome, hemophilia, and thrombocytosis; and (c) missing clinical information >15%. Only patients in the cohort from January 2019 to August 2022 were included due to the unavailability of the outcome event. Finally, 1525 eligible stroke patients were enrolled for subsequent analysis (Fig. 1). The study was approved by Zhengzhou University (Zhengzhou, China) Institutional Review Board (reference number for ethics approval: ZZUIRB2022-84).

### Baseline measurements

Body mass index (BMI) was obtained by dividing weight (kg) by the square of height (m^2^) and was classified into four groups according to Chinese health standards [19]. The participants were classified into non-smokers, former smokers and current smokers. Non-smokers were defined as never smoked or rarely smoked in the past, and have smoked continuously or cumulatively for less than 6 months. A quitter who has stopped smoking for six consecutive months was considered a former smoker. Current smokers were defined as smoking more than one cigarette per day for at least 6 months per year. Drinking status included non-drinkers, ever drinkers and current drinkers. Those who drank less than once per week in the past year were considered non-drinkers. Ever drinkers were defined as abstinence within 1 year. Current drinkers were defined as drinking more than twice per week for at least 6 months per year. Stroke subtypes were ischemic, hemorrhagic and mixed stroke. Transfusion history included prior transfusion at any time of patient’s life and acute transfusion after this admission. Previous stroke history included a history of previous ischemic stroke, hemorrhagic stroke, and transient ischemic attack. Complications and National Institutes of Health Stroke Scale (NIHSS) score were obtained from electronic health records. Thrombolysis was defined as thrombolytic therapy with urokinase, alteplase and tenecteplase. Anticoagulants mainly incorporated fondaparinux/VKA, rivaroxaban, warfarin, and low molecular weight heparin.

### Outcome assessment

The outcome was the first occurrence of VTE during hospitalization. More specifically, patients were diagnosed as DVT with intraluminal blocking or filling defects in the deep veins of the upper or lower limbs illustrated by venography or deep vein thrombogenesis evidenced by color Doppler ultrasonography. PE was confirmed with intraluminal blocking and/or filling defects in pulmonary arteries by pulmonary angiography, computed tomographic pulmonary arteriography, magnetic resonance or multiple pulmonary segmental perfusion defects shown by radionuclide lung ventilation-perfusion scans.

### Statistical analysis

A continuous variable was expressed as medians with interquartile ranges M (P25, P75) due to its abnormal distribution. Categorical variables were expressed as numbers and percentages. Multiple imputation was used to deal with missing data. The chi-square test or Mann-Whitney U test was used to compare the differences in demographic characteristics between patients with VTE and patients without VTE. Multivariate logistic regression models were used to explore the associations among transfusion history, previous stroke history and VTE.

The status of TH and PSH can be divided into four categories: a) Non-TH & Non-PSH group: no history of transfusion and previous stroke; b) TH & Non-PSH group: participants with a history of blood transfusion only; c) Non-TH & PSH group: participants with a history of previous stroke only; d) TH & PSH group: participants with both history of blood transfusion and previous stroke. Based on Virchow’s triad, we hypothesized that the joint presence of TH and PSH would potentially influence the risk of VTE in stroke patients.

According to the new STROBE statement, multiplicative and additive interaction models should be integrated when evaluating the combined impact of multiple factors [20]. For the calculation of multiplicative interaction, “TH*PSH” was included as an interaction term in the multivariate logistic regression models, and the odds ratio (OR) of “TH*PSH” was used to evaluate the significance and magnitude of multiplicative interaction. The biological interactions based on an additive scale between TH and PSH on VTE were evaluated with relative excess risk ratio (RERI), attribution percentage (AP), and synergy index (S). RERI is the excess risk attributed to interaction relative to the risk without exposure to TH and PSH. AP refers to the attributable proportion of disease caused by the interaction in subjects with both exposures. S is the excess risk from both exposures when there is a biological interaction relative to the risk from exposure to both risk factors without interaction. In the absence of additive interactions, RERI and AP equal 0 [21]. Indicative biological interactions would be considered when RERI>0, AP>0, or S>1. All estimates were adjusted by age, gender, smoking, drinking, atrial fibrillation, cancer and anticoagulants.

For subgroup analysis, we analyzed interactions according to stroke severity (measured by NIHSS score, code = 1 for NIHSS score above mean [>5 points]). All the statistical analyses were performed by SPSS 26.0 and R package version 4.1.3. *P*<0.05 (two-sided) was considered statistically significant.

## Results

### The demographics of the study population

A total of 1525 individuals were eligible in this study, with 281 (18.4%) participants who developed VTE. 734 patients (48.1%) were 60 years and older and males accounted for 62.8% of the total population. Smokers and drinkers accounted for 21.9% and 22.1%, respectively. 224 (14.7%) patients with a history of transfusion and 382 (25.1%) patients with previous stroke history. The detailed demographic characteristics are presented in Table 1.

**Table 1:** Demographics and clinical characteristics

**Characteristics**	**All subjects** **(*N* = 1525)**	**VTE**		***Z/χ***^***2***^	***P* value**
		**VTE=0** **(*N* = 1244)**	**VTE=1** **(*N* = 281)**		
Gender				6.409	0.011*
Female	567 (37.2)	444 (78.3)	123 (21.7)		
Male	958 (62.8)	800 (83.5)	158 (16.5)		
Age (years old)				20.218	<0.001*
< 60	791 (51.9)	677 (85.6)	114 (14.4)		
60 ~	555 (36.4)	436 (78.6)	119 (21.4)		
≥ 75	179 (11.7)	131 (73.2)	48 (26.8)		
BMI (kg/m^2^)				5.824	0.120
< 18.5	37 (2.4)	25 (67.6)	12 (32.4)		
18.5 ~	599 (39.3)	489 (81.6)	110 (18.4)		
24.0 ~	668 (43.8)	544 (81.4)	124 (18.6)		
≥ 28.0	221 (14.5)	186 (84.2)	35 (15.8)		
Smoking status				6.674	0.036*
Non-smokers	1059 (69.4)	846 (79.9)	213 (20.1)		
Former smokers	132 (8.7)	114 (86.4)	18 (13.6)		
Current smokers	334 (21.9)	284 (85.0)	50 (15.0)		
Drinking status				11.295	0.004*
Non-drinkers	1117 (73.2)	890 (79.7)	227 (20.3)		
Ever drinkers	71 (4.7)	65 (91.5)	6 (8.5)		
Current drinkers	337 (22.1)	289 (85.8)	48 (14.2)		
Stroke subtypes				115.937	<0.001*
Ischemic	1175 (77.1)	1023 (87.1)	152 (12.9)		
Hemorrhagic	269 (17.6)	181 (67.3)	88 (32.7)		
Both	81 (5.3)	40 (49.4)	41 (50.6)		
Transfusion history	224 (14.7)	134 (59.8)	90 (40.2)	82.654	<0.001*
Previous stroke history	382 (25.1)	294 (77.0)	88 (23.0)	7.207	0.007*
Family history of stroke	243 (15.9)	202 (83.1)	41 (16.9)	0.464	0.496
Diabetes mellitus	450 (29.5)	360 (80.0)	90 (20.0)	1.052	0.305
Hypertension	1058 (69.4)	862 (81.5)	196 (18.5)	0.023	0.880
Hyperlipidemia	182 (11.9)	154 (84.6)	28 (15.4)	1.272	0.259
Atrial fibrillation	78 (5.1)	47 (60.3)	31 (39.7)	24.853	<0.001*
Cancer	37 (2.4)	25 (67.6)	12 (32.4)	4.949	0.026*
Anticoagulants	358 (23.5)	208 (58.1)	150 (41.9)	171.490	<0.001*
Thrombolysis	134 (8.8)	105 (78.4)	29 (21.6)	1.011	0.315
NIHSS score	3 (2, 7)	3 (1, 6)	7 (3, 18)	-11.162	<0.001*

Values are presented as median (interquartile range) or n (%)

^*^P<0.05 was considered statistically significant

### Association of TH and PSH with stroke

With the incidence of VTE as the dependent variable, univariate logistic regression analysis was performed with TH or PSH as independent variables, respectively. The results showed that TH and PSH were statistically significant with the risk of VTE (*P*<0.05). The presence of TH led to a 3.903 (95% CI: 2.868~5.312, *P*<0.001) times risk of VTE than the absence of TH. Patients with PSH also had a 1.473 (95% CI: 1.109~1.957, *P*<0.007) times risk of VTE than non-PSH subjects. Then TH, PSH, age, gender, smoking, drinking, atrial fibrillation, cancer and anticoagulants were incorporated into the logistic regression model for multivariate analysis. We found that TH and PSH were still significantly associated with VTE in stroke patients (*P*<0.05, Table 2).

**Table 2:** Logistic regression analysis of TH, PSH and VTE in stroke patients

**Variables**	**Univariate analysis**		**Multivariate analysis**	
	**OR (95% CI)**	***P***	**OR (95% CI)**	***P***
TH				
No	Ref	—	Ref	—
Yes	3.903 (2.868, 5.312)	<0.001*	2.296 (1.576, 3.345)	<0.001*
PSH				
No	Ref	—	Ref	—
Yes	1.473 (1.109, 1.957)	0.007*	1.702 (1.211, 2.394)	0.002*

Multivariate analysis were adjusted by age, gender, smoking, drinking, atrial fibrillation, cancer, and anticoagulants

*P<0.05 was considered statistically significant

—No data

### Interaction effects of TH and PSH on VTE in stroke patients

To investigate the effect of coexistent TH and PSH on VTE, we divided subjects into four categories (non‐TH & non‐PSH, TH & non‐PSH, non‐TH & PSH, and TH & PSH) (Table 3). In the unadjusted model, TH and PSH group showed 11.166 (95% CI: 5.759~21.647, *P*<0.001) times risk than the non‐TH and non‐PSH group for VTE. After adjusting covariates, all of the effect values attenuated but remained significant. TH and PSH group showed a 7.096 (3.300~15.260, *P*<0.001) times risk than the non-TH & non-PSH group, much higher than that of TH & non‐PSH group (1.868, 95% CI: 1.224~2.852, *P*=0.004) and non‐TH & PSH group (1.436, 95% CI: 0.983~2.096, *P*=0.061). However, we found that Non-TH & PSH group was significantly associated with the incidence of VTE in the unadjusted model but not yet significant in the adjusted model.

**Table 3:** The multiplicative and additive interaction between TH and PSH on VTE in stroke patients

**Variables**	**VTE**	**No VTE**	**Unadjusted model**		**Adjusted model**^**a**^	
			**OR (95% CI)**	***P***	**OR (95% CI)**	***P***
**Multiplicative scale**						
TH	90 (40.2)	134 (59.8)	3.465 (2.427, 4.946)	<0.001*	1.868 (1.224, 2.852)	0.004*
PSH	88 (23.0)	294 (77.0)	1.432 (1.027, 1.995)	0.034*	1.436 (0.983, 2.096)	0.061
TH * PSH	26 (63.4)	15 (36.6)	2.251 (1.033, 4.905)	0.041*	2.646 (1.080, 6.481)	0.033*
**Additive scale**						
Non-TH & Non-PSH	129 (13.4)	831 (86.6)	Ref	—	Ref	—
TH & Non-PSH	64 (35.0)	119 (65.0)	3.465 (2.427, 4.946)	<0.001*	1.868 (1.224, 2.852)	0.004*
Non-TH & PSH	62 (18.2)	279 (81.8)	1.432 (1.027, 1.995)	0.034*	1.436 (0.983, 2.096)	0.061
TH & PSH	26 (63.4)	15 (36.6)	11.166 (5.759, 21.647)	<0.001*	7.096 (3.300, 15.260)	<0.001*
RERI	—	—	7.270 (1.891, 17.606)	—	7.016 (1.489, 18.165)	—
AP	—	—	0.651 (0.260, 0.793)	—	0.650 (0.204, 0.797)	—
S	—	—	3.510 (1.568, 7.860)	—	3.529 (1.451, 8.579)	—

^*^P<0.05 was considered statistically significant

^a^Adjusted by age, gender, smoking, drinking, atrial fibrillation, cancer, and anticoagulants

—No data

**Fig. 2:**
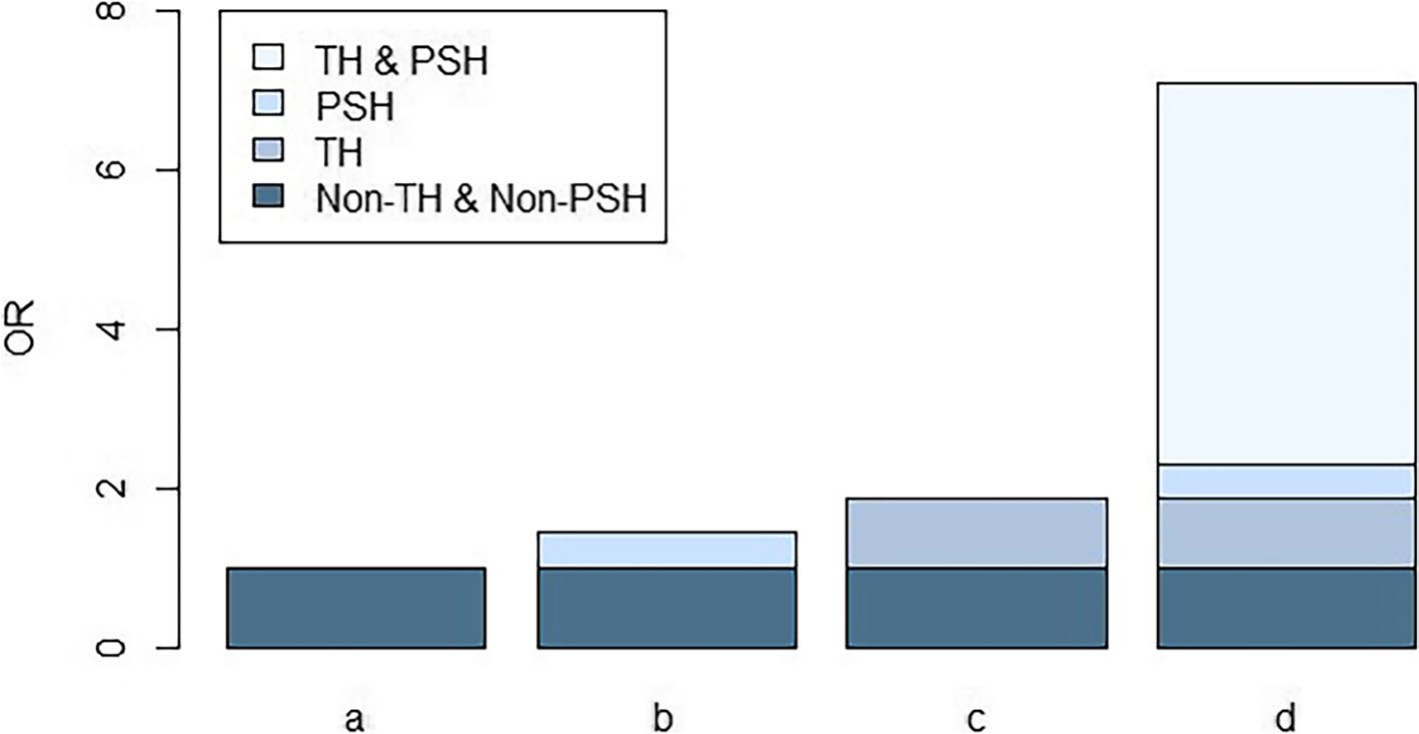
OR of VTE with contributions from different exposure. Note: Abbreviations: OR, Odds ratio; TH, transfusion history; PSH, previous stroke history. Non-TH & Non-PSH: *n*=960; TH & Non-PSH: *n*=183; Non-TH & PSH: *n*=341; TH & PSH: *n*=41

Table 3 also presents the interaction effect in both additive and multiplicative scales. The interaction effect measured in the multiplicative scale showed statistical significance towards VTE in both unadjusted and adjusted models (OR=2.251, 95% CI: 1.033~4.905 vs. OR=2.646, 95% CI: 1.080~6.481; *P*<0.05). For the additive scale, the RERI (7.270, 95% CI: 1.891~17.606) for VTE was significant and positive in the unadjusted model. After adjusting covariates, the RERI shrank to 7.016 (95% CI: 1.489~18.165), demonstrating the significant and synergistic interaction between TH and PSH towards VTE. Furthermore, the AP for VTE revealed that 65.0% of the total risk was attributed to the synergistic interaction between TH and PSH. Moreover, the S index of VTE was 3.529 (95% CI: 1.451~8.579), indicating the synergistic interaction between TH and PSH. Measures quantifying interaction on an additive scale suggested a supra-additive effect of the combination of TH and PSH on the risk of VTE (Fig. 2).

We then divided our population into two subgroups by NIHSS score and re-evaluated the interaction effect in both scales (Table 4). In participants with NIHSS score ≤ 5 points, the interaction was neither significant on VTE in the multiplicative nor the additive scale. While the addictive interaction was significant in patients with NIHSS score > 5 points, accounting for 75.6% and 74.4% of the unadjusted and adjusted model, respectively. Additionally, measures quantifying interaction on an additive scale demonstrated a supra-additive effect of the combination of TH and PSH on the risk of VTE (Fig. S1 in the supplement). As for the multiplicative interaction, the results were consistent with our primary results but more significant than the whole population (Table 4).

**Table 4:** Subgroup analysis

**Variables**	**VTE**	**No VTE**	**Unadjusted**		**Adjusted**^**a**^	
			**OR (95% CI)**	***P***	**OR (95% CI)**	***P***
**NIHSS score ≤ 5 points**						
**Multiplicative scale**						
TH	26 (28.9)	64 (71.1)	3.516 (1.962, 6.299)	<0.001*	1.814 (0.891, 3.693)	0.101
PSH	39 (15.4)	214 (84.6)	1.589 (1.008, 2.504)	0.046*	1.423 (0.835, 2.426)	0.195
TH * PSH	7 (43.7)	9 (56.3)	1.417 (0.424, 4.734)	0.571	2.205 (0.518, 9.391)	0.285
**Additive scale**						
Non-TH & Non-PSH	92 (9.0)	631 (91.0)	Ref	—	Ref	—
TH & Non-PSH	19 (25.7)	55 (74.3)	3.516 (1.962, 6.299)	<0.001*	1.814 (0.891, 3.693)	0.101
Non-TH & PSH	32 (13.5)	205 (86.5)	1.589 (1.008, 2.504)	0.046*	1.423 (0.835, 2.426)	0.195
TH & PSH	7 (43.7)	9 (56.3)	7.916 (2.850, 21.987)	<0.001*	5.692 (1.663, 19.487)	0.006*
RERI	—	—	3.811 (-1.754, 17.733)	—	3.455 (-0.887, 17.133)	—
AP	—	—	0.481 (-0.688, 0.713)	—	0.607 (-0.773, 0.772)	—
S	—	—	2.228 (0.609, 8.144)	—	3.793 (0.605, 23.759)	—
**NIHSS score > 5 points**						
**Multiplicative scale**						
TH	64 (47.8)	70 (52.2)	2.099 (1.310, 3.362)	0.002*	1.523 (0.888, 2.612)	0.126
PSH	49 (38.0)	80 (62.0)	1.210 (0.729, 2.008)	0.460	1.399 (0.792, 2.470)	0.247
TH * PSH	19 (76.0)	6 (24.0)	3.722 (1.220, 11.355)	<0.021*	3.529 (1.027, 12.122)	0.045*
**Additive scale**						
Non-TH & Non-PSH	67 (25.1)	200 (74.9)	Ref	—	Ref	—
TH & Non-PSH	45 (41.3)	64 (58.7)	2.099 (1.310, 3.362)	0.002*	1.523 (0.888, 2.612)	0.126
Non-TH & PSH	30 (28.9)	74 (71.1)	1.210 (0.729, 2.008)	0.460	1.399 (0.792, 2.470)	0.247
TH & PSH	19 (76.0)	6 (24.0)	9.453 (3.624, 24.654)	<0.001*	7.519 (2.607, 21.685)	<0.001*
RERI	—	—	7.144 (1.409, 22.170)	—	5.597 (0.735, 19.593)	—
AP	—	—	0.756 (0.236, 0.846)	—	0.744 (0.066, 0.849)	—
S	—	—	6.457 (1.731, 24.087)	—	7.069 (1.344, 37.192)	—

^*^P<0.05 was considered statistically significant

^a^Adjusted by age, gender, smoking, drinking, atrial fibrillation, cancer, and anticoagulants

—No data

## Discussion

Our study for the first time found that the joint exposure of TH and PSH may yield a positive multiplicative and supra-additive effect on the risk of VTE based on a prospective Chinese stroke cohort. And the present results were consistent with our initial hypothesis. The interaction effect measured in the multiplicative scale showed statistical significance towards VTE after adjustment (OR=2.646, 95% CI: 1.080~6.481, *P*<0.05). Individuals exposed to both TH and PSH had a 7-fold higher risk of VTE compared to individuals exposed to neither risk factors on an additive scale, and the combined effect of the two exposures exceeded the sum of the independent effects. Accordingly, 65% of the VTE events occurring among study participants jointly exposed to TH and PSH were attributed to the interaction between the two risk factors. In subgroup analyses, compared with patients with an NIHSS score < 5 points, the interaction effect was pronounced for the risk of VTE in severe stroke patients (NIHSS score > 5 points).

Previous research on the joint effect of TH and PSH on VTE is very limited. However, the independent effect of TH and PSH on VTE is supported by substantial evidence. Several studies have demonstrated that acute transfusions are associated with the risk of VTE, which are consistent with our findings. A retrospective cohort study suggested that postoperative blood transfusions may be associated with an increased risk of postoperative VTE, independent of confounders [6]. Acuña et al. [22] found that the receipt of a postoperative transfusion was relevant to an increased risk of VTE in 333, 463 patients with total knee arthroplasty. Furthermore, another single-center retrospective cohort study also confirmed that the risk of VTE following total joint arthroplasty increased by approximately three-fold when blood transfusions were prescribed [7]. Patients often receive acute transfusion during the perioperative period. Increasing evidence suggests the role of red blood cells and platelet in physiological hemostasis and pathologic thrombosis [9, 23]. Studies have shown that plasma transfusion can lead to a significant increase in fibrinogen and soluble clotting factors (II, V, VII, IX, X) and caused hypercoagulability [24]. In addition, red blood cells are associated with inflammatory cascade, altered tissue perfusion, impaired vasoregulation and storage lesions, all of which may promote prothrombogenic [25]. Due to the close relationship between hypercoagulability and inflammation, the proinflammatory and immunomodulatory abilities of red blood cells transfusion may further accelerate the hypercoagulable status [26]. Additionally, studies have revealed that red blood cells could participate in thrombosis by increasing platelet reactivity and mediating platelet adhesion to the intact endothelial surface [27].

Established evidence has stated a correlation between prior transfusion and VTE risk. A case-crossover study [28] found that a history of transfusion during one year or earlier was a significant predictor of VTE, with an incidence rate ratio of 2.57 (*P*=0.018) after adjustment for covariates. A multicentre propensity score matching study showed that transfusion had a negative impact on long-term survival in patients with early stages of perihilar cholangiocarcinoma treated after curative resection [29]. Worse long-term survival may be associated with infection and immobility, which are risk factors of VTE. In addition, patients with multiple blood transfusions were chronically exposed to foreign antigens, which may be associated with abnormalities of immunologic function [30]. Previous evidence also indicated that immune cells and inflammatory processes are involved in VTE initiation [31]. Therefore, it is reasonable to speculate that blood transfusion may have long-term effects on cell and coagulation states in the body. The adverse reactions of transfusion, such as immune inflammatory reactions and antibodies, can remain in the patient’s body and be activated at any time. Their ability to affect coagulation is multifactorial. It may depend on their mechanical properties potentially affecting vascular endothelium, molecular signaling via microvesicles and surface proteins, including blood group antigens, immunomodulation, altered tissue perfusion, impaired vasoregulation, and participation in nitric oxide metabolism [32]. There is evidence that the transfused blood cells have stronger adhesion to endothelial cells and exacerbate microvascular pathology [33]. In summary, both prior and acute blood transfusions may be associated with VTE risk. However, we did not collect data on composition and frequency of blood transfusion, the number of transfused red blood cells, platelets or plasma, which will be the focus of our future research.

Evidence also supports the association between prior stroke and VTE. Neurological deficits following stroke frequently contribute to immobility and predispose to common complications such as VTE [34]. A population-based study of 2483 central Massachusetts residents in America suggested that patients with a history of prior stroke were more likely to have provoked VTE than those without stroke (*P*<0.001) [11]. The underlying mechanism may be owing to the hypercoagulation status of blood and paralysis associated with the sequelae in prior stroke patients [11, 12].

The potential mechanism behind such interaction is still unclear. However, as both TH and PSH are associated with hypercoagulability and inflammatory response. One might speculate that the observed excess VTE risk could be related to increased blood viscosity and inflammation [7, 35, 36]. Thus, it is likely to speculate that a biological interaction between TH and PSH on the risk of VTE could be mediated via procoagulant changes arising from two sources. Moreover, PSH is also associated with subsequent dysfunction of extremities, such as paralysis and long-term bed rest, which are common risk factors of VTE, and potentially lead to increased risk of VTE [37]. However, the specific mechanism remains unclear, further study is still warranted.

Interestingly, several studies suggested that VTE appears to occur more frequently in non-smokers and non-drinkers, which supports the results of our study. A large cohort study with 144,952 participants indicated that smoking was associated with increased VTE risk in cancer subjects, but not in non-cancer subjects [17]. A recent meta-analysis of ten prospective studies (1 441,128 individuals) also showed that compared with the lowest group, the highest consumption of alcohol was not associated with the VTE risk (*P*=0.293) [38]. Interestingly, a large case-control study indicated that alcohol consumption was associated with a reduced risk of VTE [39]. However, further studies with larger samples are needed to validate the results.

In subgroup analyses, the interaction effect measured in the multiplicative and additive scale was significant for severe stroke patients with NIHSS score > 5 points. The possible explanation is that severe stroke patients may have higher blood viscosity, which further aggravates the hypercoagulability of blood caused by TH and PSH [24]. Besides, limb dysfunction and prolonged immobility associated with severe stroke may further increase this association.

Considering the strong and synergistic interaction between TH and PSH on VTE in stroke patients, it is essential to implement measures to prevent and treat VTE in those patients. The combination of TH and PSH could aid in the screening process to identify high-risk populations before VTE and may contribute to more effective prevention of VTE in clinical practice. For stroke patients with TH and PSH, health education on VTE prevention was necessary for them and their families. Additionally, clinicians can give corresponding physical intervention, such as intermittent pneumatic compression and thrombus elastic stocking, and anticoagulant therapy to those patients.

The current study has several strengths. First, our participants were derived from a large prospective Chinese stroke cohort and the data can also be linked to electronic health records, which was accurate and reliable. Second, both multiplicative and additive interactions were evaluated, and the results were consistent in theory, which provided strong support for the main conclusion. Additionally, our study for the first time implicated the interaction effect between TH and PSH towards VTE in stroke patients, which may provide a new perspective and reference for thromboprophylaxis in stroke patients.

Meanwhile, there are still several limitations to our study. First, we can only provide a clue for the synergistic interaction between TH and PSH towards VTE, but the causality of the association between the synergistic interaction and VTE still needs more prospective studies to verify. Second, our findings were derived from Chinese stroke subjects, which may not be generalized to other ethnic populations. Thirdly, we did not collect data on composition and frequency, number of blood transfusions, and the quantity number of transfused red blood cells, platelets or plasma, the variables associated with pre-stroke disability such as the Rankin score or Barthel score, which should be paid attention to in future research. In addition, patients in some subgroups were too few, which may reduce the statistical power of the interaction. The results of this study still need to be validated in a larger sample size prospective cohort study. Finally, as in any observational study, we cannot exclude the potential presence of unrecognized residual confounding and inevitable selection bias. However, patients with prior stroke and prior transfusion were not prioritized for investigation. Therefore, extensive studies with more potential moderators are warranted.

## Conclusions

In conclusion, our study suggested a significant interaction effect between TH and PSH on the risk of VTE in stroke patients. A substantial proportion of VTE was attributable to the interaction. Besides, the percentage of VTE incidence explained by interaction increased with the severity of stroke. Overall, these findings will provide valuable evidence for the combined effects of TH and PSH in predicting VTE in Chinese stroke patients. Future studies are warranted to verify our findings and explore the specific biological mechanisms of interaction.

## Supplementary Information

**Additional file 1:** **Figure S1.** OR of VTE from different exposure in patients with NIHSS score > 5 points. 

## Data Availability

The datasets used and/or analyzed during the current study are available from the corresponding author upon reasonable request.

## Abbreviations

AP: Attributable proportion
NIHSS: National Institutes of Health Stroke Scale
OR: Odds ratio
PSH: Previous stroke history
RERI: Relative excess risk of interaction
S: Synergy index
TH: Transfusion history
VTE: Venous thromboembolism
