# Supplementary material for: The interaction effect of transfusion history and previous stroke history on the risk of venous thromboembolism in stroke patients: a prospective cohort study
Source: Thromb J. 2023 Apr 17;21:41. doi: 10.1186/s12959-023-00487-2 (PMC10108449; doi:10.1186/s12959-023-00487-2)
Supplement: Supplementary file 1 — Additional file 1: Figure S1. OR of VTE from different exposure in patients with NIHSS score > 5 points. [file 12959_2023_487_MOESM1_ESM.docx]

# Supplementary materials

Figure S1 OR of VTE from different exposure in patients with NIHSS score > 5 points


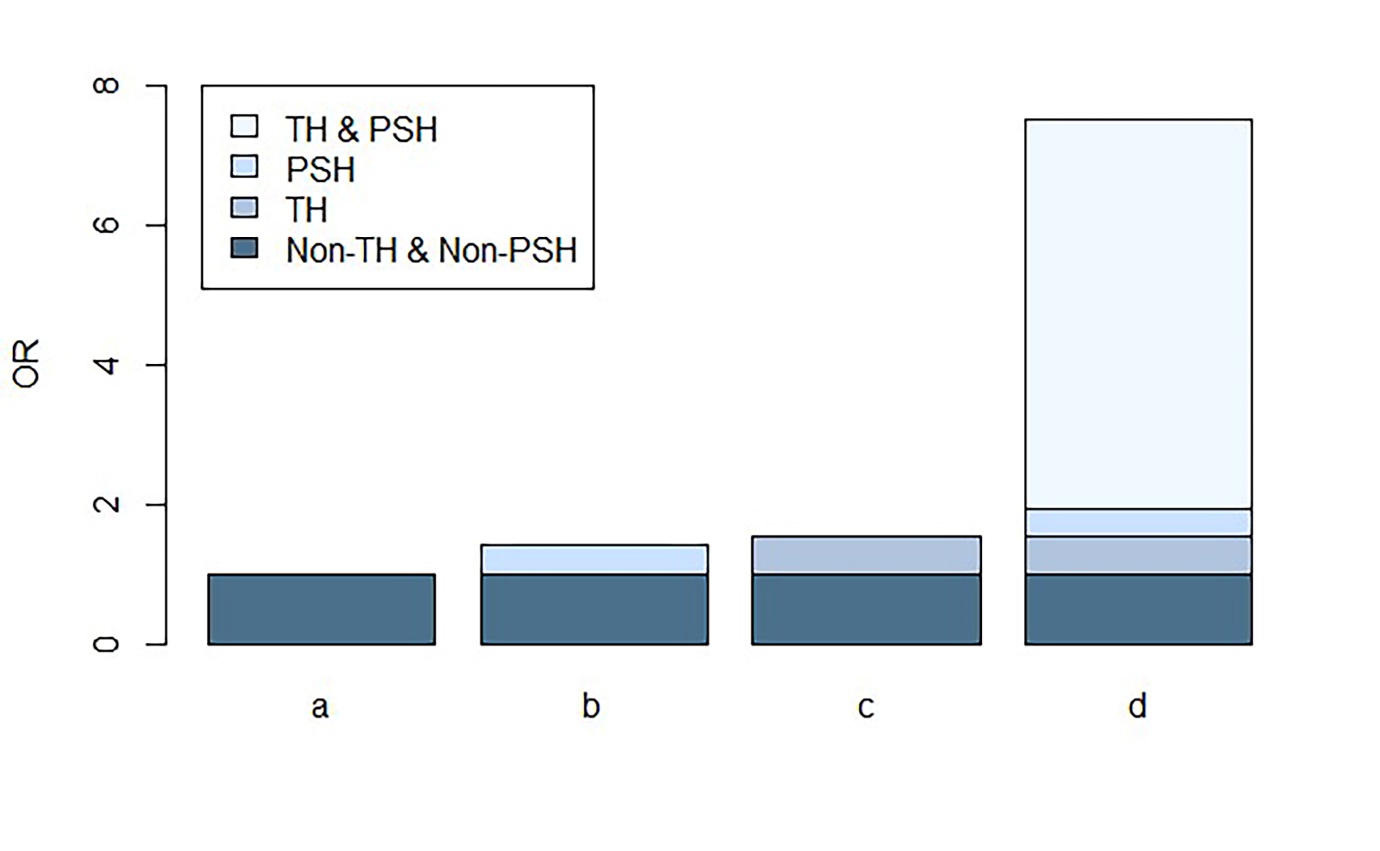


**Note:** Abbreviations: OR, Odds ratio; TH, transfusion history; PSH, previous stroke history. Non-TH & Non-PSH: n=267; TH & Non-PSH: n=109; Non-TH & PSH: n=104; TH & PSH: n=25.
